# Understanding motivating and demotivating factors among maternal healthcare professionals in Somalia: a qualitative interview study

**DOI:** 10.1136/bmjgh-2024-018479

**Published:** 2026-02-24

**Authors:** Naima Said Sheikh, Abdi Gele, Igna Bonfrer

**Affiliations:** 1 Department of Public Health Science, Norwegian University of Life Sciences, As, Akershus, Norway; 2 Somali Institute for Health Research, Garowe, Somalia; 3 Erasmus University Rotterdam, Rotterdam, The Netherlands

**Keywords:** Global Health, Health policy, Health systems, Maternal health, Qualitative study

## Abstract

**Introduction:**

Motivated health workers are pivotal in providing adequate health services. This study aims to understand what motivates and demotivates maternal health workers. We do so in Somalia, an understudied country in Africa with pervasive security challenges and one of the highest avoidable maternal mortality rates.

**Methods:**

This qualitative study explores health workers’ motivation in three tertiary hospitals in the capital, Mogadishu. Twenty skilled healthcare professionals were interviewed, including nurses, midwives, physicians, specialists and supervisors. The interviews were transcribed verbatim and analysed using thematic analysis.

**Results:**

Key factors influencing healthcare workers’ motivation include job satisfaction, monetary and work-related support, effective managerial practices, career development and intrinsic motivation. Most health workers expressed a powerful combination of altruism, volunteerism and religious conviction, driving their professional commitment to the community. Challenges that led to demotivation included high patient volume, staff shortages, limited supplies, infrastructural constraints, unregulated managerial practices and health system limitations. While most health workers primarily wanted to meet patients’ needs and did not consider salary a decisive motivating factor, others were demotivated by low pay and heavy workload.

**Conclusion:**

Maternal health workers in Somalia face challenges that impact their motivation. Mitigating burnout through workload management and continued education can contribute to a more motivated and resilient healthcare workforce. Policy recommendations include offering long-term contracts, providing access to training and implementing fair and transparent employment policies. Further research is needed to evaluate the effectiveness of both financial and non-financial incentives in motivating health workers in Somalia.

**WHAT IS ALREADY KNOWN ON THIS TOPIC**

- Maternal mortality remains a critical public health issue in the Global South, particularly in sub-Saharan Africa, where healthcare workers often experience poor motivation due to factors such as inadequate pay and challenging working conditions.
- Somali midwives and gynaecologists, especially females, report low levels of motivation due to high workload and limited resources.

**WHAT THIS STUDY ADDS**

- This study provides an in-depth understanding of the factors influencing the motivation of Somali maternal health workers, including career development, heavy workload, lack of supplies and shortages of skilled health professionals.
- Most health workers expressed a powerful combination of altruism, volunteerism and religious conviction, driving their professional commitment to the community.

**HOW THIS STUDY MIGHT AFFECT RESEARCH, PRACTICE AND POLICY**

- Addressing challenges in staff shortages, limited supplies and management practices while enhancing intrinsic and extrinsic incentives is likely to create a more motivated and effective healthcare workforce.
- We suggest targeted interventions, including non-financial incentives such as increased opportunities for education and career development for healthcare workers, accompanied by an impact evaluation to determine what works in increasing motivation and in turn quality of maternal care.

## Introduction

 Maternal mortality is a major public health concern in the Global South, where women and young girls face a much higher risk of dying from avoidable pregnancy and childbirth complications.^1 2^ The causes of maternal and infant mortalities in the Global South are multifaceted and complex, associated with multiple challenges, including access and availability of skilled healthcare professionals.^1 3 4^ Many health workers in low-income countries face challenges, including poor working conditions, staff shortages, limited trained staff and low motivation,^5^,^8^ which can negatively impact performance and service delivery.^9^ Motivated health workers are pivotal for providing adequate health services and improving population health,^10^ especially in sub-Saharan Africa (SSA).^11^ However, only a few studies investigate health worker motivation in SSA.^12^,^17^

Health worker motivation reflects the health workers’ willingness to exert and maintain an effort towards attaining organisational goals.^18^ This motivation is linked to both financial and non-financial rewards. Fritzen argues that so-called daylight factors, including managerial supervision, job security, salary and the opportunity for training and promotion, have a major impact on health workers’ motivation and behaviour.^19^ Other ‘shadow’ factors such as social expectations, professional ethics, alternative sources of income, informal organisational culture and expectations also affect health worker motivation. Furthermore, motivation can be differentiated into intrinsic and extrinsic. Intrinsic motivation refers to internal forces that motivate humans, in this context health workers, to meet their personal and organisational aims. Intrinsically motivated health providers display desirable behaviours or attitudes towards patients.^20^ Extrinsic motivation refers to external factors: ‘doing something with the purpose of obtaining a separable outcome’.^21^ The latter type of motivation includes aspects such as working conditions, security, promotion and material benefits such as payments, rewards or other bonuses and awards, as well as symbolic elements such as recognition, commendation and opportunities for continuing education.^22 23^ Motivation is also associated with work attitudes such as job satisfaction and organisational commitment.^24 25^ Job satisfaction is an individual’s emotional reaction and behavioural expression of their work achievement and environment.^25^ Dieleman *et al*^12^ state that ‘two different areas of motivation are often confused: motivation to be in a job and to perform’, adding that both are important in the work setting. Motivation is the main driver behind the transformation of capacity (ie, the measurable output from infrastructure and supplies) into performance, and health workers who are motivated to provide high levels of effort also generally have high levels of performance when provided enough capacity.^22^

Empirical studies from low and middle-income countries (LMICs) highlight diverse motivational dynamics. In Ethiopia, motivation was higher among health workers in private hospitals than in public hospitals, with both intrinsic and extrinsic factors shaping their motivation.^26^ Demotivating factors included delayed salaries, resource shortages, excessive workloads, limited training opportunities and poor organisational leadership.^27^ In Nigeria, motivation among primary healthcare workers was linked to feeling supported, recognised and included in a fair and collaborative work environment, as well as organisational practices and a strong culture of teamwork,^28^ while others reported motivating factors to include feelings of confidence, acceptance, happiness, a sense of identity and receiving a monthly payment.^29^ Furthermore, a study from Zambia found that health worker motivation was higher among those who were older, had received recent training or had longer job tenure; notably, higher scores of motivation were related to conscientiousness and timeliness.^30^ In Nepal, the majority of health workers employed by the local government reported job satisfaction.^31^

### Study setting

Somalia has not been able to establish a robust health system due to three decades of civil war, limiting access to basic healthcare. Maternal mortality is at 693 per 100 000 births,^32^ the third highest in the world and the country faces a severe shortage of maternal health workers. Gynaecologists and midwives report lower motivation than other health workers.^14^ The impact of multitasking, a high-intensity workspace and high expectations from leadership and patients might have led to some degree of physical or emotional exhaustion and subsequent lack of motivation for midwives and gynaecologists.^14^ This study aimed to describe and understand what motivates and demotivates maternal healthcare workers in their everyday work. Conducted in Somalia, a very understudied country with one of the highest avoidable maternal mortality rates, this qualitative study complements our earlier quantitative research on health worker motivation in Somalia.^14^ To the best of our knowledge, this is the first study of its kind to qualitatively explore the motivation of the maternal health workforce in Somalia and the challenges they encounter.

## Methods

### Study design and setting

This qualitative study was conducted between May and July 2020 using in-depth interviews with 20 health workers. A semi-structured, open-ended approach was used to allow participants flexibility in their responses and share their experiences, providing deeper insights into their motivation. Due to COVID-19 restrictions, the interviews were conducted digitally via Zoom and lasted 30–50 min. Participants were purposively selected from three prominent hospitals in Somalia: Benadir Maternity and Children Hospital, Somali-Turkish Training and Research Hospital and SOS Hospital. These hospitals are among the country’s most prominent and best-equipped public healthcare institutions, attracting many patients and healthcare workers. We selected a diverse sample of professionals, including nurses, midwives, physicians, paediatricians, gynaecologists and a supervisor, as all of these were directly or indirectly involved in the maternal and child health service. Given the critical shortage of healthcare professionals and limited resources in the country, each of these roles plays an essential role in supporting maternal and child health. Including this broad range of workers allowed us to capture varied perspectives on motivation and challenges across different functions and levels of responsibility within the maternal healthcare system.

### Recruitment and data collection

Participants were recruited in collaboration with hospital administration, including department directors. A digital meeting was held to explain the study aim and the relevance of improving maternal healthcare services. Based on staff registries, we employed purposive sampling to identify eligible participants who worked exclusively in maternal and child health departments.

Although we aimed to include a more diverse range of professionals, the sample size of 20 was based on data saturation, as participants did not provide new themes despite being asked probing questions. We also followed the guidelines for selecting the correct sample size.^33 34^

The interview guide was based on validated items developed by Mbindyo *et al*^13^ and was designed to be triangulated with quantitative methods to examine the same elements studied in our earlier study.^14^ The qualitative method gives participants not only an opportunity to respond in their own words but also allows researchers to probe participants’ initial responses.^35^ Participants were asked about working conditions, salary, workloads, burnouts, commitment to the hospitals and job satisfaction and were probed to identify other intrinsic and extrinsic motivating factors. All interviews were audio-recorded with the participants’ consent, transcribed verbatim in Somali and then translated into English for analysis.

### Data analysis

This study was conducted and reported in accordance with the Standards for Reporting Qualitative Research (see online supplemental file 1). The first author, NSS, conducted, transcribed and analysed all interviews using NVivo V.12 and Microsoft Word. The recording was subsequently replayed to check the transcript for consistency. The authors, NSS and AG, carefully read the transcripts for accuracy and completeness. Data were anonymised before analysis, and all identifiers were removed, so identifying the health workers who participated in the study was impossible for anyone other than the interviewer. The narratives and recurring patterns from the participants formed the basis for coding and analysing the raw data. Data were analysed using inductive thematic analysis, following the process described by Braun and Clarke.^36^ The coded excerpts and quotations were reviewed to help understand the link between different concepts and develop core themes and subthemes, with reliability verified through cooperative reflection by authors NSS and AG. The thematic analysis allowed the research findings to emerge naturally from the participants’ interviews without the restrictions that might be created by more structured methodologies.^37^ The first author’s academic background (Nursing) and cultural familiarity within the context of the study may have influenced the interpretation of the data. However, ongoing discussions and reflections with the coauthors were maintained.

## Results

### Sociodemographic characteristics

Table 1 shows sample characteristics of the participants. Out of the 20 health workers interviewed, the majority were under 30 years of age and had diverse educational backgrounds, from high school diploma to paediatrics specialisation. All physicians had at least 6 years of higher education, with specialists having obtained an additional 4 years of training. Nurses and midwives completed at least a bachelor’s degree in their respective fields.

**Table 1:** Sociodemographic and professional characteristics of study participants (n=20)

	Total participants
Sex	11 females
	9 males
Age	Ranged from 25 to 51 years
	Mean: 29.0 years
Work experience	Ranged from 9 months to 27 years
	Mean work experience: approximately 5 years
Educational background	9 Bachelor of Medicine and Surgery
	2 paediatric specialists
	3 Bachelor of Nursing
	2 Bachelor of Nursing and Midwifery specialist
	2 Bachelor of Nursing and master’s in public health
	1 Bachelor of Medicine and master’s in public health
	1 high school diploma
Positions	Physicians
	Nurses
	Midwives
	Paediatricians
	Supervisor

**Figure 1:**
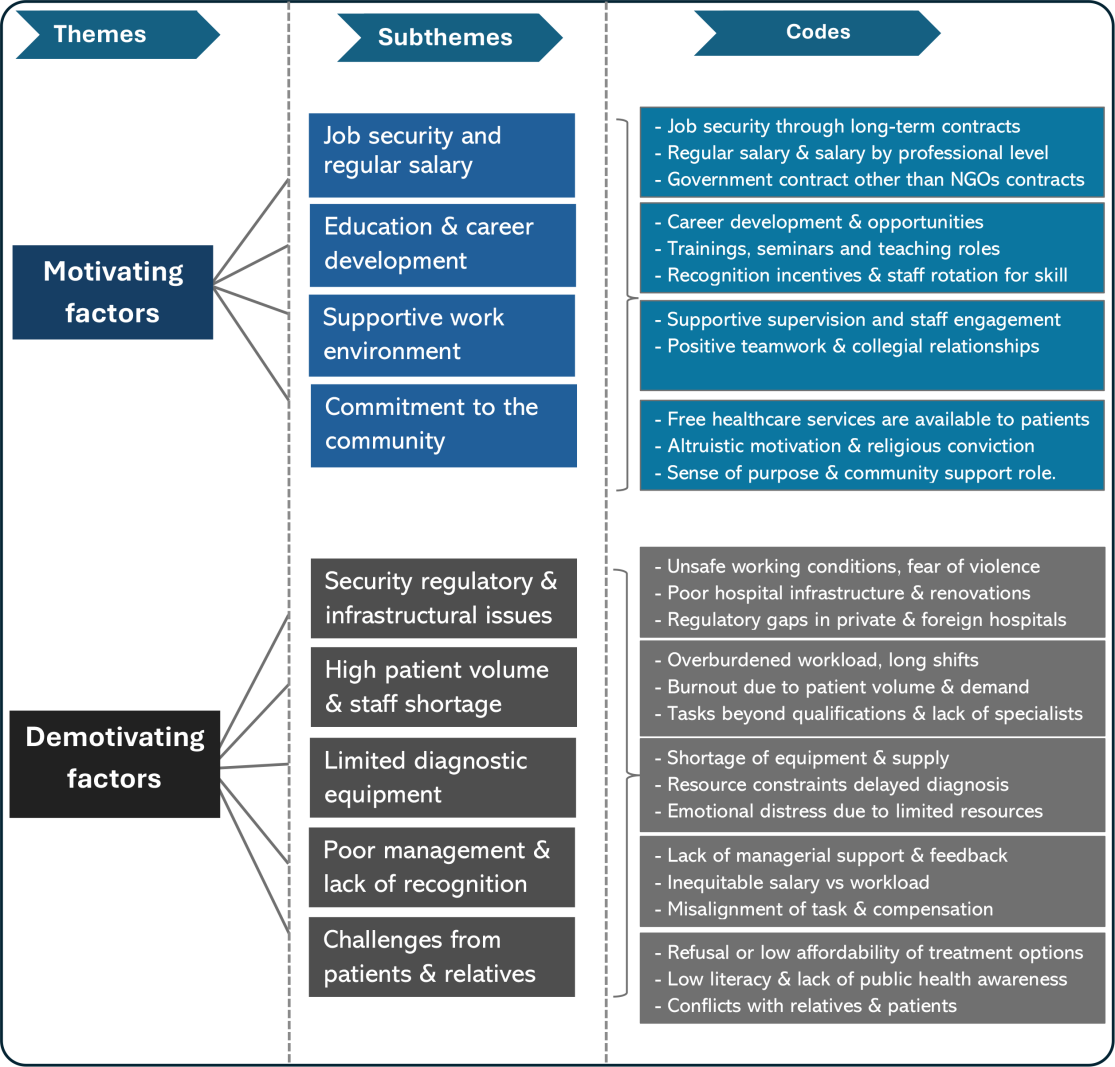
Overview of thematic categories and subthemes related to health worker motivation among maternal healthcare workers in Somalia.

As shown in figure 1, this study identified two main themes: motivating and demotivating factors, and nine subthemes, including job satisfaction, monetary and work-related support, good managerial practices, career development and other intrinsic motivations. Challenges related to working conditions and health system limitations were identified as demotivating factors.

### Motivating factors

#### Job security and regular salary

Most health workers mentioned job security, including long-term contracts and regular salary, as motivating factors, with many expressing satisfaction with their current salary. Those with government contracts received better benefits and a more reliable salary than those with non-governmental (NGO) contracts, who expressed concerns about lower monetary support. Salary payments were based on their academic level, with an average monthly salary for nurses of US$400–600, physicians US$800–1000 and specialists of US$2000−2500. Additionally, some health workers interviewed had been working as unpaid volunteers for years, despite being academically qualified, in the hope of gaining experience and skills that could potentially lead to permanent and paid employment at the hospitals.

Since the government overtook the hospital’s responsibility, we receive regular salaries, which is much better than the NGO’s. But only a few health workers have government contracts. (Respondent 1, male, physician)

#### Education and professional growth

Education and career development were identified as motivators for most health workers interviewed. The aspiration for professional progression was evident not only in the perceived value attached to undergoing training and seminar sessions but also in the recognition of gaining incentives, such as prizes and certificates, as a mechanism for enhanced engagement.

I am thankful that they gave us chances for further specialisations, and since the hospital is a teaching hospital, they trained us to teach other medical students. I also have a contract to work as a paediatric specialist for three years. (Respondent 15, male, paediatric specialist)

A nurse suggested a proactive approach to professional development and operational efficiency in healthcare settings by rotating staff to different departments.

I would suggest that the hospital send us to different departments every three weeks so we can increase our skills and work experience in every department. This will benefit patients and the hospital, so we can work and help other departments when there is a staff shortage. (Respondent 9, female, nurse)

#### Supportive work environment

Most health workers stated that a positive and supportive work environment is crucial for maintaining motivation. Furthermore, health workers indicated that they had good relationships with colleagues and supervisors and identified that as a vital motivating factor, emphasising the significance of encouraging teamwork. Other health workers acknowledged the encouragement from the senior staff members and managerial support as necessary for enhancing motivation, especially when problems arise.

We work together as a team and cooperate well. Everyone has specific responsibilities. We prioritise the patient and work. If something goes wrong, we inform the administration, and things get fixed, so they motivate us to do our work. (Respondent 1, male, physician)

#### Commitment to serving the community

Most health workers expressed a powerful combination of altruism, volunteerism and religious conviction, driving their professional commitment to the community. Most health workers interviewed mentioned that their motivation is due to their strong commitment and dedication to the community they serve. They perceived that their role had significance for the patients, gave a sense of purpose and supplemented the government’s efforts to fill the gaps in public services.

My motivation is to help the people. We are here to support the hospital and the community (…). Before I worked here as a volunteer for many years, I was not interested in having a salary. Allah (God) wants us to help those who suffer. (Respondent 4, female, nurse & midwife)

### Demotivating factors

#### High patient volume and staff shortages

Most health workers stated that overburdening workloads, long shifts and challenging work conditions negatively influenced their motivation. They indicated that excessive workload led to burnout due to pressure and frustrations resulting from a high number of patients per shift and performing tasks beyond their qualifications. Limited human resources, particularly the shortage of highly skilled specialists for complex cases, were recognised as a major issue that affected the quality of care provided, particularly during outbreaks such as COVID-19, cholera and other infectious diseases.

At least 900 women give birth monthly in our hospital, and we receive almost 100 new patients daily. Sometimes, there might be 3 to 5 staff in the department and less qualified specialists, so we have to perform challenging tasks which give us frustrations. (Respondent 11, female physician)

There are problems related to limited specialists. We receive patients daily, and due to challenges in treatment options and resources, we sometimes send patients back to their homes or transfer them to other hospitals. It is tough to see patients who need treatments, and due to limited resources, we cannot help them. (Respondent 8, female nurse)

#### Limited diagnostic and medical equipment

Shortages or lack of essential medical supplies and diagnostic materials were reported as demotivators, affecting the ability to provide timely and effective diagnoses and treatments. Most of them stated that the struggle with shortages of medical equipment and necessary materials was most notable in intensive care units (ICU) for neonatal care and departments with patients in critical conditions. Several interviewees indicated that these shortages led to undiagnosed cases, patient dissatisfaction, lower prioritisation of critically ill patients and emotional distress for health workers.

We have a shortage of equipment and supplies, especially in the ICU and the neonatal care unit. I think the biggest problem in our healthcare system is the availability of hospital resources. Some patients cannot have surgery due to a lack of ICU supplies. (Respondent 7, female, nurse)

#### Poor management and lack of recognition

Some health workers expressed dissatisfaction with task alignment, salary, leave policies and hospital managerial involvement. Those with longer shifts and greater responsibilities felt their salaries did not reflect their workload, which varied daily. Hospital administrations were criticised for failing to balance workload with staff capacity. Additionally, the lack of recognition, appreciation and opportunities for career development and self-improvement contributed to their demotivation. Health workers also reported receiving infrequent feedback and limited access to seminars and training, as a supervisor noted a lack of support from the hospital management.

We rarely see the management of the hospital. It would be nice if they came to us, talked to us, and showed some recognition and appreciation. I wish they provided some training and seminars. (Respondent 13, female, midwife)

Unfortunately, I received no support or appreciation from the hospital management. I was the only supervisor for three departments with over 200 patients and their staff for almost two months. (Respondent 10, female, supervisor)

Dissatisfaction with leave policies for maternity leave, sickness leave and holidays was identified as a demotivating factor, especially for females who receive fewer leave options than those working at public institutions.

When we asked for maternity leave, they gave us eight weeks only and told us that they wanted to reduce female assistants and cleaners because of them getting pregnant and asking for maternity leave. It is not fair (Respondent 2, female, physician).

#### Disparities in patients’ financial access and refusal of treatment options

Health workers stated that financial barriers and socioeconomic disparities patients face, which often prevent necessary treatments, influence the motivation to provide care. Most health workers mentioned that patients and their relatives frequently refused treatments and held negative expectations of health workers, causing emotional distress and demotivation. Health workers noted that challenges arise when patients and their families refuse essential treatments, leading to adverse health outcomes. Some health workers explained that limited understanding or low health literacy led patients to choose specific treatment options out of fear or misconceptions about alternatives. Most health workers emphasised the need for public education campaigns on the importance of timely medical treatments and the role of health providers in the decision-making process.

Some patients delay hospital visits due to the costs of health interventions. … Sometimes, it is terrifying because we don’t know what kind of patient they are. Are they good or bad people? Will they cooperate ? We are always on high alert to avoid problems. (Respondent 14, male, pediatric specialist)

Most Somali people are illiterate. If a young female needs a cesarean section (CS) to give birth, family members refuse, and sometimes the baby dies, or both the baby and the mother die. (Respondent 17, male, physician)

#### Infrastructural constraints, security and regulatory issues

Poor hospital infrastructure was also mentioned as a demotivating factor. Some health workers reported that outdated hospital buildings and a lack of hospital renovation contributed to discomfort among patients and health professionals, affecting their motivation. Most health workers noted that hospitals need infrastructure improvements and that public hospitals need to be rebuilt.

The hospital was built in 1966, and few changes have been made since then. Patients sometimes say they would prefer to stay home due to the conditions at the hospital. They do not feel comfortable staying in the hospital. The hospital needs improvements. (Respondent 1, male, physician)

Fear of terror attacks, kidnappings and other forms of violence was reported as a demotivator. Some health workers stated that the pervasive security challenges in Mogadishu had significantly impacted their safety, work schedules and ability to provide care, as well as the risks associated with long commutes and the necessity to travel during unsafe hours. A supervisor mentioned that some health workers encountered violence, some were killed and female staff had to use face covers outside the hospital to protect their identities.

We had security problems; even some of the staff members were killed We always experience some harassment and fear. Most of our female staff use face covering outside the hospital to hide their identity. (Respondent 10, female, supervisor)

The absence of quality control and regulatory policies in the healthcare system, particularly policies concerning private hospitals and foreign doctors working in Somalia, was highlighted as a demotivating factor. Health workers mentioned the regulatory gap where the qualifications and skills of healthcare providers, especially foreign health professionals, are not adequately inspected, and that they received patients with severe complications, and even patients died due to treatments provided by private hospitals.

We receive patients who experienced complications after being admitted to those private hospitals. To get a job in public hospitals, the academic and practical skills are tested before they hire new health workers. I suggest that the Ministry of Health control foreign doctors who come to Somalia. (Respondent 11, female, midwife)

## Discussion

The findings of this study contribute to the understanding of the motivation and challenges faced by maternal healthcare workers in Somalia. The primary motivating factors included job satisfaction, financial and work-related support, good managerial practices, career development opportunities and intrinsic motivation. Among the aspects of demotivation identified, high patient volume, staff shortages, limited supply, infrastructural constraints, managerial policies and health system limitations were described as crucial challenges.

### Motivating factors

Non-financial incentives such as education, career development and job satisfaction were considered essential motivation factors. Health workers mentioned that access to further education and career development, which were comparatively more accessible for those with government contracts, influenced their motivation. Earlier work on health worker motivation in Somalia found that higher education was associated with increased motivation.^14^ This finding is consistent with a study from Kenya, which argued that continuous improvements in formal and informal knowledge are essential for motivation and achieving institutional goals, with main benefits including improved self-esteem, job security, job satisfaction, refreshing past knowledge and practices and identifying and correcting mistakes.^38^ Additionally, studies reported that career development, appreciation, training opportunities and good working conditions were considered motivating factors for health workers.^39 40^ Despite irregularities in the workforce at privately owned hospitals, which is widespread in Somalia, some health workers noted that government employment contracts were necessary to receive regular benefits, including a regular and sufficient salary. However, this type of contract was deemed primarily relevant for those in higher education with more experience in the field.

Health workers experienced good management practices, positive relationships with colleagues and supervisors, and supportive, committed management as key motivating factors. These findings align with results among health workers in East Africa.^41^ Recognition and collaboration within the department team were viewed as instrumental in fostering a positive work environment and motivation. A study reported that supportive supervision improves professional development, improves health workers’ job satisfaction and increases motivation.^42^,^44^ Health workers’ motivation increases when managers provide a clear sense of vision and mission, involve health workers in decision-making, give feedback and create opportunities for promotion.^45^,^47^

Regarding financial incentives, some health workers considered monetary support, including salary and allowances, to be less important. In contrast, others were dissatisfied with their salaries due to the workload and responsibilities they have at the hospitals. Earlier work showed that volunteers with no monetary support from the workplace were more motivated than those who received a salary.^14^ This could be explained by the expectation of being employed at the hospitals after their volunteering period. In Rwanda, among the list of motivators, the monetary determinants ranked lower in the list compared with non-monetary determinants,^41^ while Pakistani physicians and Malian health workers ranked salary and good pay as the first and the second important motivating factor, respectively.^48^ While salary is a determinant of employee motivation, it is not the only motivating factor for workers to perform better^48 49^ and does not enhance their intrinsic motivation.^50^ In line with our findings, health workers value both financial and non-financial rewards and may accept lower salaries if other job aspects are attractive.^51^ In Somalia, many health workers prioritised contributing positively to the community, believing in the meaningfulness of their roles for the community and expressing the motivation to help others. Such results of altruism were reported in a study among health workers in East Africa, that their determinant of motivation resulted from the intrinsic desire to help others, that is, family, community, or the public.^41^

### Demotivating factors

Health workers identified several demotivating factors, including high patient volume and staff shortages, limited diagnostic and medical equipment, poor management practices and health system limitations. Overburdening and unmanageable workloads, long shifts, bad work conditions and emotional distress contributed to burnout and demotivation, exacerbating the shortages of skilled professionals. A review study on the motivation of health workers in LMICs found that increased workload and staff shortages have been associated with greater levels of burnout, higher rates of injuries, intent to leave the job and more frequent absenteeism.^52^ Besides, heavy workloads may cause stress, fatigue and tiredness, negatively affecting motivation.^53^ Similarly, limited diagnostic materials and inadequate medical supplies hinder timely and effective treatments, also demotivating health workers in Somalia. Similar results were reported in East African countries, whereas shortages or lack of essential medical supplies and equipment demotivate health workers.^41^ Consequently, limited human resources and staffing in Somalia have led to difficulties in meeting patient expectations, refusal of treatment options and fear of negative consequences from treatments received at the hospitals.

Health workers mentioned poor hospital management, lack of recognition and task misalignment as demotivators. They expressed frustrations over the lack of recognition and appreciation from the hospital management, limited opportunities for self-improvement and career development, and not receiving feedback. A study from Iran reported similar demotivators, which included poor management, lack of recognition and appreciation, subjective performance appraisal, lack of job description and difficult living conditions.^48^ Additionally, healthcare system limitations, infrastructural constraints, safety challenges and the absence of proper regulations for healthcare providers were mentioned as demotivating factors. Security concerns, long commutes and travelling during unsafe hours in Mogadishu had significantly impacted safety, work schedules and ability to provide care. Besides, the unregulated private healthcare system worsens the already existing challenges in Somalia, with issues such as poor patient-provider relationships, inappropriate treatment, unnecessary laboratory tests, excessive use of advanced diagnostic technologies and overcharging.^54^

### Gendered patterns in motivation

Although gender differences were not the primary focus of this study, certain patterns emerged that may explain our previous findings that female healthcare workers were less motivated than their male counterparts.^14^ Female respondents expressed specific concerns related to maternity leave, institutional discrimination and personal safety, with some describing how they felt undervalued. In contrast, male health workers more often emphasised opportunities for professional development, such as specialisation and leadership roles. These observations are consistent with evidence from SSA, where around 70% of community health workers are young women, most of whom work without pay, and only 43% received any form of non-financial incentives.^55^ This may reflect broader gender imbalances in health system leadership, as only 38% of ministries of health in Africa are led by women.^55^ Overall, these findings suggest that gendered dynamics influence how motivation is experienced in the maternal healthcare workforce and highlight the need for a gender-sensitive approach in future research and health system planning.

### Strengths and limitations

This exploratory qualitative study aimed to enhance our understanding of patterns underlying health workers’ motivation in conflict settings like Somalia. A key strength of this study is its focus on a critically understudied setting, offering context-specific insights that can inform targeted intervention in Somalia’s maternal health sector. Participants were recruited from three major hospitals and diverse departments (eg, outpatient and inpatient care units, antenatal care, postnatal care, delivery department, ICU) with diverse education levels and roles (from nurses to department supervisors). These variations increase the transferability of the findings to similar settings. To support the trustworthiness of the study, we followed established qualitative research procedures, including systematic approaches to data collection and analysis, while continuously reflecting on our perspectives and potential biases through the research process. However, this study has some limitations. First, the relatively small size of 20 participants may limit the breadth of perspectives. However, it was sufficient to achieve the thematic analysis required for this study. Second, some interviews were conducted during the COVID-19 pandemic and some during work shifts, which may have introduced some bias. Third, recall bias may have influenced participants’ ability to accurately reflect on past experiences, while social desirability bias may have led some to overstate positive aspects of their motivation and job satisfaction. Fourth, while purposive sampling helped ensure diversity, sampling bias cannot be entirely ruled out, as those more willing or available to participate may differ systematically from those who decline to participate. Finally, the study was limited to the perspectives of health workers from nurses to department supervisors. The uneven distribution of participants across professional categories reflects the actual composition of the team during the data collection period. We did not include views and perspectives from hospital managers and policymakers.

## Conclusion

This study identifies key motivating and demotivating factors among maternal health workers in Somalia, highlighting the multifaceted challenges that might influence their working conditions and overall motivation. Both intrinsic and extrinsic incentives can improve the quality of care provided without increasing the capacity of clinicians. For example, enhancing access to training, strengthening institutional communication and fostering intrinsic motivation are essential for increasing job satisfaction. At the same time, managing workload, promoting continued education and professional development can contribute to a more motivated and resilient healthcare workforce. Hence, a policy recommendation includes offering long-term contracts, providing access to training and implementing fair and transparent employment policies to boost motivation and job satisfaction. Additionally, increasing the supply of equipment and drugs and enhancing professionalism through education and supportive supervision can further improve the quality of health services provided. Future research is needed to assess the effectiveness of different financial and non-financial incentives in motivating health workers in Somalia.

## Supplementary material

10.1136/bmjgh-2024-018479 online supplemental file 1

## Data Availability

Data are available upon reasonable request.

## References

[1] Abejirinde IOO, Ilozumba O, Marchal B (2018). Mobile health and the performance of maternal health care workers in low- and middle-income countries: A realist review. Int J Care Coord.

[2] Chou D, Daelmans B, Jolivet RR (2015). Ending preventable maternal and newborn mortality and stillbirths. BMJ.

[3] Merriel A, Dembo Z, Hussein J (2021). Assessing the impact of a motivational intervention to improve the working lives of maternity healthcare workers: a quantitative and qualitative evaluation of a feasibility study in Malawi. Pilot Feasibility Stud.

[4] Abraham JM, Melendez-Torres GJ (2023). A realist review of interventions targeting maternal health in low- and middle-income countries. Womens Health (Lond).

[5] World Health Organization (2016). Midwives voices, midwives realities. findings from a global consultation on providing quality midwifery care. https://iris.who.int/handle/10665/250376.

[6] Mere RA, Simbeni TV, Mathibe M (2023). Job satisfaction among health professionals in a District of North West province, South Africa. Health SA.

[7] Milku ND, Abose DW, Gelaw KA (2024). Challenges and coping strategies for providing maternal health care services among health care professionals in rural health facilities in Wolaita Zone, Southern Ethiopia: a qualitative study. BMC Health Serv Res.

[8] Adatara P, Amooba PA, Afaya A (2021). Challenges experienced by midwives working in rural communities in the Upper East Region of Ghana: a qualitative study. BMC Pregnancy Childbirth.

[9] Friedman EA, Bickford R, Bjork C (2023). The global health and care worker compact: evidence base and policy considerations. BMJ Glob Health.

[10] Chowdhury M, Meena USJ, Barker P (2023). A motivated workforce is needed for quality improvement efforts to succeed. BMJ.

[11] Ahmat A, Okoroafor SC, Kazanga I (2022). The health workforce status in the WHO African Region: findings of a cross-sectional study. BMJ Glob Health.

[12] Dieleman M, Toonen J, Touré H (2006). The match between motivation and performance management of health sector workers in Mali. Hum Resour Health.

[13] Mbindyo PM, Blaauw D, Gilson L (2009). Developing a tool to measure health worker motivation in district hospitals in Kenya. Hum Resour Health.

[14] Sheikh NS, Gele A (2023). Factors influencing the motivation of maternal health workers in conflict setting of Mogadishu, Somalia. *PLOS Glob Public Health*.

[15] Olaniran A, Madaj B, Bar-Zeev S (2022). Factors influencing motivation and job satisfaction of community health workers in Africa and Asia-A multi-country study. Int J Health Plann Manage.

[16] Ndambo MK, Munyaneza F, Aron MB (2022). Qualitative assessment of community health workers’ perspective on their motivation in community-based primary health care in rural Malawi. BMC Health Serv Res.

[17] Ejigu Y, Abera N, Haileselassie W (2023). Motivation and job satisfaction of community health workers in Ethiopia: a mixed-methods approach. Hum Resour Health.

[18] Franco LM, Bennett S, Kanfer R (2002). Health sector reform and public sector health worker motivation: a conceptual framework. Soc Sci Med.

[19] Fritzen SA (2007). Strategic management of the health workforce in developing countries: what have we learned?. Hum Resour Health.

[20] Lagarde M, Huicho L, Papanicolas I (2019). Motivating provision of high quality care: it is not all about the money. BMJ.

[21] Ryan RM, Deci EL (2000). Intrinsic and Extrinsic Motivations: Classic Definitions and New Directions. Contemp Educ Psychol.

[22] Leonard KL, Masatu MC, Herbst CH (2015). The systematic assessment of health worker performance. http://hdl.handle.net/10986/24076.

[23] Baljoon RA, Banjar HE, Banakhar MA (2018). Nurses’ Work Motivation and the Factors Affecting It: A Scoping Review. *Int J Nurs Clin Pract*.

[24] Aduo-Adjei K, Emmanuel O, Forster OM (2016). The Impact of Motivation on the Work Performance of Health Workers (Korle Bu Teaching Hospital): Evidence from Ghana. *Hosp Pract Res*.

[25] Tharikh SM, Ying CY, Mohamed Saad Z (2016). Managing Job Attitudes: The Roles of Job Satisfaction and Organizational Commitment on Organizational Citizenship Behaviors. Procedia Economics and Finance.

[26] Ayichew G, Atnafu DD, Hussein M (2025). Health workers’ motivation was significantly higher in private hospitals than public hospitals influenced by intrinsic and extrinsic factors in Northwest Ethiopia. Front Public Health.

[27] Milkano TM, Daka K, Bolado GN (2025). Job motivation and associated factors among health workers providing maternal and child health services in Wolaita Zone public hospitals, Southern Ethiopia; A mixed-method study. PLoS One.

[28] Ebenso B, Mbachu C, Etiaba E (2020). Which mechanisms explain motivation the of primary health workers? Insights from the realist evaluation of a maternal and child health programme in Nigeria. BMJ Glob Health.

[29] Mbachu C, Etiaba E, Ebenso B (2022). Village health worker motivation for better performance in a maternal and child health programme in Nigeria: A realist evaluation. J Health Serv Res Policy.

[30] Mutale W, Ayles H, Bond V (2013). Measuring health workers’ motivation in rural health facilities: baseline results from three study districts in Zambia. Hum Resour Health.

[31] Poudel S, Kaphle HP, Parajuli S (2025). Work motivation and job satisfaction among local government health workers in Chitwan, Nepal: a cross-sectional study. BMJPH.

[32] UNFPA Somalia (2020). The somali health and demographic survey 2020. https://somalia.unfpa.org/en/publications/somali-health-and-demographic-survey-2020.

[33] Hennink M, Kaiser BN (2022). Sample sizes for saturation in qualitative research: A systematic review of empirical tests. Soc Sci Med.

[34] Wutich A, Beresford M, Bernard HR (2024). Sample Sizes for 10 Types of Qualitative Data Analysis: An Integrative Review, Empirical Guidance, and Next Steps. Int J Qual Methods.

[35] Mack N, Woodsong C, Macqueen KM (2005). Qualitative research methods: A data collector’s field guide.

[36] Braun V, Clarke V (2006). Using thematic analysis in psychology. Qual Res Psychol.

[37] Hay I, Cope M, Hay I (2021). Qualitative research methods in human geography.

[38] Momanyi GO, Adoyo MA, Mwangi EM (2016). Value of training on motivation among health workers in Narok County, Kenya. Pan Afr Med J.

[39] Margie N, Artati NE, Rusmawati A (2023). The Influence of Career Development and Compensation through Motivation on the Performance of Healthcare Workers in Buaran and Kedungwuni Sub-District Community Health Center. *Saudi J Econ Fin*.

[40] D’Arrietta LM, Vangaveti VN, Crowe MJ (2024). Exploring the motivation of health professionals to engage with research at various career stages. BMC Health Serv Res.

[41] Muthuri R, Senkubuge F, Hongoro C (2020). Determinants of Motivation among Healthcare Workers in the East African Community between 2009–2019: A Systematic Review. Healthcare (Basel).

[42] Edosomwan HS, Nwanzu CL, Omreore OE (2024). Relationship between Supportive Supervision and Caring Behavior among Healthcare Workers: The Mediating Role of Job Satisfaction. Int J Occup Saf Health.

[43] Madede T, Sidat M, McAuliffe E (2017). The impact of a supportive supervision intervention on health workers in Niassa, Mozambique: a cluster-controlled trial. Hum Resour Health.

[44] Hotchkiss DR, Banteyerga H, Tharaney M (2015). Job satisfaction and motivation among public sector health workers: evidence from Ethiopia. Hum Resour Health.

[45] Kok MC, Vallières F, Tulloch O (2018). Does supportive supervision enhance community health worker motivation? A mixed-methods study in four African countries. Health Policy Plan.

[46] Grant C, Nawal D, Guntur SM (2018). “We pledge to improve the health of our entire community”: Improving health worker motivation and performance in Bihar, India through teamwork, recognition, and non-financial incentives. PLoS ONE.

[47] Okello DRO, Gilson L (2015). Exploring the influence of trust relationships on motivation in the health sector: a systematic review. Hum Resour Health.

[48] Daneshkohan A, Zarei E, Mansouri T (2025). Factors Affecting Job Motivation among Health Workers: A Study From Iran. GJHS.

[49] Alase G, Akinbo T (2021). Employee Motivation and Job Performance: Empirical Evidence from Nigeria. AJMSS.

[50] Olafsen AH, Halvari H, Forest J (2015). Show them the money? The role of pay, managerial need support, and justice in a self‐determination theory model of intrinsic work motivation. Scandinavian J Psychology.

[51] Hongoro C, Normand C, Jamison DT, Breman JG, Measham AR (2006). Disease control priorities in developing countries.

[52] Assaye AM, Wiechula R, Schultz TJ (2021). Impact of nurse staffing on patient and nurse workforce outcomes in acute care settings in low- and middle-income countries: a systematic review. JBI Evid Synth.

[53] Rusli B, Lee MY, Ekhsan B (2019). The impact of workload on job performance among doctors in Malaysian public hospitals. a case study. https://www.semanticscholar.org/paper/The-Impact-Of-Workload-On-Job-Performance-Among-In-Rusli-Lee/0a3d72be999bd5430787ec355e2d11e6d35a2717.

[54] Gele AA, Ahmed MY, Kour P (2017). Beneficiaries of conflict: a qualitative study of people’s trust in the private health care system in Mogadishu, Somalia. Risk Manag Healthc Policy.

[55] Holt T, Sun Y (2024). Sub-saharan africa’s healthcare worker shortage. https://www.mckinsey.com/industries/social-sector/our-insights/overcoming-sub-saharan-africas-health-workforce-paradox.

